# Increase in Reported Legionellosis — Milwaukee, Wisconsin, June–September 2013

**Published:** 2014-01-24

**Authors:** Paul A. Biedrzycki, Marisa Stanley, Fredrick Radmer, Shannon Lauf

**Affiliations:** 1 City of Milwaukee Health Department

In early July 2013, the City of Milwaukee Health Department (MHD) was notified by the Wisconsin Division of Public Health of an increase in reported cases of legionellosis in southeastern Wisconsin. Legionellosis is a reportable disease to state and local public health authorities in Wisconsin. During June 1–September 30, 2013, a total of 58 clinically diagnosed cases of Legionnaires’ disease, confirmed by laboratory testing, were reported in Milwaukee County, more than twice the number of total annual case reports in each of the previous 5 years. Forty-five (78%) of these cases were reported in the city of Milwaukee. The median age of county patients was 53 years (range = 29–77 years); all but one was hospitalized, and no deaths were reported. MHD received one report of a death attributed to legionellosis in the county during this period.

Environmental sampling for detection of *Legionella* was initiated by MHD at 11 sites within the city of Milwaukee, including select commercial building cooling towers; a large, decorative, outdoor water fountain; a public swimming pool/waterpark with spray features; and two residences of homebound patients. Thirty-nine swab and bulk water specimens were collected. Samples taken from three different cooling towers were positive for *Legionella pneumophila* serogroup 1, but were not genetically matched. Pulsed-field gel electrophoresis performed by the Wisconsin State Laboratory of Hygiene and MHD on nine lower-respiratory specimens from confirmed cases and tests by the MHD laboratory at the 11 environmental sites revealed six distinct strains of *L. pneumophila*. No strains found in the patients were related to the strains found in environmental samples.

Mapping of county cases using spatial analysis software showed that 31 (53%) patients reported home addresses within 3 miles of one or more of the three *Legionella*-positive towers. In comparison, approximately 40% of the county’s residents live within 3 miles of one or more of the three geographically separate towers. Although a relative risk of 1.6 indicated increased risk for *Legionella* exposure within a 3-mile radius of a tower, the association was not statistically significant (95% confidence interval = 0.9–2.7).

To address the risk for *Legionella* exposure, MHD met with regional cooling tower contractors and consultants. Gaps were identified in seasonal cooling tower maintenance and operation caused by unseasonably cool June weather followed by extremely hot weather in early July 2013. This resulted in a delayed start of building air conditioning operation after a prolonged period of tower disuse. In response, MHD distributed the CDC Procedure for Cleaning Towers Infected with Legionella* to local building maintenance organizations, realty management groups, and heating, ventilation, and air conditioning professional organizations.

The increase in legionellosis in the city of Milwaukee during June–September 2013 suggests that cases were community-acquired from multiple environmental sources, possibly including contaminated cooling towers. Multiple physician alerts issued by MHD and DPH in July 2013 led to an increase in testing for *Legionella* by providers. Consequently, heightened clinician and laboratory surveillance might have contributed to a portion of the recorded increase in legionellosis. This investigation underscores the need for local public health authorities to be prepared to rapidly enhance surveillance, deliver appropriate public risk messaging, and coordinate with the private sector to mitigate environmental transmission of *Legionella* within a community.

## Footnotes

* Available at http://city.milwaukee.gov/imagelibrary/groups/healthauthors/dcp/pdfs/cdcprocedureforcleaningtowerle.pdf.

